# EmpowermentPLUS: Dismantling Stigma for Families Affected by Younger‐Onset Dementia

**DOI:** 10.1002/alz70858_101169

**Published:** 2025-12-25

**Authors:** Diana Shulla Cose

**Affiliations:** ^1^ Lorenzo's House, Chicago, IL, USA

## Abstract

Through the **EmpowermentPlus Model (Model)**, we envision a world where daughters, sons and their families walking with younger‐onset dementia ultimately end the stigma cycle and drive dementia justice. We have two goals for the Model. First, we are training our young people in the EmpowermentPlus Model so that they are able to dismantle dementia stigma in their own lives, and train and support others to do the same–a youth‐led train‐the‐trainer model. Secondly, we aim to dismantle dementia stigma locally, nationally and globally by sharing the Model through our speaking engagements, social media and publications. Our young people who have a parent with younger‐onset are the teachers and the compass for ALL who are working to bring awareness to lives with dementia. We envision a world where those living with brain change and their families are embraced and where dementia is not stigmatized.

Specifically, youth caregivers learn and teach others to utilize the EmpowermentPLUS Model during our annual SUMMIT and continue to utilize the Model during CLUBS. Our youth caregivers then dismantle stigma with their person living with younger‐onset dementia in their own homes and in their communities.

**EmpowermentPLUS Model steps are as follows**:

**RECOGNIZE** when we experience stigma Notice how this experience feels Define what this experience means

**ACCESS** personal & community toolkit with courage and intention Identify technique from toolkit Determine how to respond

**EMPOWER** self & others by educating & dismantling stigma Respond with empathy for self and others Reflect upon the situation

